# Properties of Yogurts Enriched with Crude Polysaccharides Extracted from *Pleurotus ostreatus* Cultivated Mushroom

**DOI:** 10.3390/foods12214033

**Published:** 2023-11-05

**Authors:** Wojciech Radzki, Katarzyna Skrzypczak, Bartosz Sołowiej, Ewa Jabłońska-Ryś, Waldemar Gustaw

**Affiliations:** 1 Department of Fruits, Vegetables and Mushrooms Technology, Faculty of Food Sciences and Biotechnology, University of Life Sciences in Lublin, Skromna 8, 20-704 Lublin, Poland; katarzyna.skrzypczak@up.lublin.pl (K.S.); ewa.jablonska-rys@up.lublin.pl (E.J.-R.); waldemar.gustaw@up.lublin.pl (W.G.); 2 Department of Dairy Technology and Functional Foods, Faculty of Food Sciences and Biotechnology, University of Life Sciences in Lublin, Skromna 8, 20-704 Lublin, Poland; bartosz.solowiej@up.lublin.pl

**Keywords:** fortified yoghurt, β-glucan, antioxidant, polysaccharide, *Pleurotus ostreatus*, functional food, texture profile analysis

## Abstract

Increasingly, consumers are looking for products with specific nutritional and health-promoting properties. The answer of the producers for this demand is fortified food. The raw material that can be used to enrich food is, among others, mushrooms. Crude water soluble polysaccharides (cWSP) were isolated from fruiting bodies of *Pleurotus ostreatus* (oyster) mushroom. Chemical analysis showed that they consisted mainly of carbohydrates (~61%), protein (~9%) and phenolics (~0.8%). The isolated cWSP were used to obtain enriched cow milk set yogurts. cWSP were added at the concentration of 0.1%, 0.2%, 0.3%, 0.4% and 0.5%, and milk containing no cWSP was prepared as the control. All of the variants were fermented via applying two commercially available culture starters. The addition of cWSP led to a drop in pH in the case of one starter culture. Also, the decline in total soluble solids (TSS) content was higher where cWSP was used for the enrichment. Texture profile analysis (TPA) revealed that parameters of hardness and gumminess increased along with the concentration of cWSP (reaching values approximately 7–8 times higher, compared to the control). A significant increase in syneresis level (proportional to cWSP concentration and ranging from ~10% to ~50%) was also observed after the fermentation. Fortifying milk with cWSP led to a slight increase in antioxidant capacity in FRAP assay (up to ~12%) and ABTS assay (up to ~23%). The results demonstrate that using cWSP to enrich set-type yogurts is fairly limited.

## 1. Introduction

In recent decades consumer demand has been shifting towards healthy food which is capable of reducing the risk of developing chronic diseases such as cardiovascular diseases, diabetes or cancer. Products or raw materials which provide additional beneficial influence on the human body beyond nutritional value have been recognized as functional food [1]. The dynamic development of the functional food market which has led to the availability of new products is currently observed [2]. These products can be produced using a wide range of raw materials, like cereal (e.g., bread and pasta), dairy (cheese, yogurts and cheese analogues), fruits and vegetables (juices, drinks and spreads), seafood or meat [1]. Among dairy functional products, probiotic yogurts have gained high consumer acceptance due to sensory attributes, convenience of use and the presence of health-promoting compounds [3]. Additionally, yogurts fortified with different plant-based health beneficial ingredients have appeared on the market or have been the subject of research. Among additives aimed at enriching yogurts, various raw materials are used, such as green tea decoction [4], flavonoid-rich extracts from grape seeds, pomegranate skins and seeds [5,6], *Aloe vera* gel [7], cherry pulp [8], fungi-derived constituents [9,10], whey proteins [11] or chia seeds [12].

Fruiting bodies of edible mushrooms contain a wide range of bioactive compounds and are considered to be functional food [13,14]. The health-promoting properties of mushrooms are associated with the presence of anticancer, anti-inflammatory, immunomodulatory, antiviral, antioxidant, prebiotic and cardioprotective compounds [15,16]. As a consequence, mushrooms themselves, as well as mushroom-derived constituents, are also used for the production of foodstuffs [17,18]. Both wild and cultivated mushrooms are a rich source of, among others, group B vitamins, phenolic compounds, terpenes, selenium or vitamin D (if fruiting bodies are exposed to sunlight or artificial UV light) [19,20]. Among health-promoting compounds, special attention is paid to bioactive polysaccharides belonging mainly to β-glucans. These high molecular weight water-soluble molecules exert antioxidant and antitumor activity, boost immune system, act as prebiotic and are capable of lowering cholesterol levels [21,22,23,24]. Additionally, they are not digested and absorbed by the human digestive system and are regarded as soluble fiber [25]. An example of such molecule is pleuran—a high molecular mass β-(1,3)-(1,6) branched glucan isolated from *Pleurotus ostreatus* (oyster mushroom) [26]. Apart from high molecular weight pleuran, other bioactive polysaccharides are present in *P. ostreatus* fruiting bodies. There are reports of lower in mass immunomodulating glucogalactans [27], antioxidant glycans [28], heterogalactans [29] and antiproliferative heteroglucans [30].

Mushroom-derived polysaccharides or polysaccharidic fractions were tested as potential food additives in different dairy matrices including yogurts [9,31], spreadable cheese [32] or processed cheese [33]. Quite often, beneficial effects were observed by the researchers. Kondyli et al. [32] reported increased moisture of spreadable cheese enriched with *P. ostreatus* β-glucans. Also, these β-glucans improved textural properties when added to white brined cheese [34]. Other authors noticed that polysaccharides from *P. ostreatus* and *Lentinula edodes* (Shiitake) had a rather neutral impact on sensory properties of yogurts and were recommended as potential food additives [35]. Also, as reported previously, water extract from *P. ostreatus* (which contains polysaccharides) improved the viscosity of yogurts as well as slightly decreased syneresis [9]. The influence of non-mushroom polysaccharide on yogurt quality has been also the subject of many studies. Experiments on some natural source polysaccharides (carrageenan, xanthan gum an alginate) carried out by [36] demonstrated the varied impacts of these molecules. While carrageenan (at the concentration 0.3–0.5%) increased firmness and water holding capacity, xanthan gum had rather little impact on texture parameters. However, the decrease in firmness was noticed when alginate was applied. Other authors reported that carrageenan and xanthan gum improved yogurt gel strength at a very low dosage but also increased syneresis and led to the decrease in gel stability [37].

The aim of this study was to produce yogurts containing different concentrations of oyster mushroom crude polysaccharides which were fermented by two commercially available starter cultures. Subsequently, this study aimed to analyze them in terms of textural, physicochemical and antioxidant properties. It was hypothesized that crude polysaccharides extracted from mushroom fruiting bodies, when added to milk prior to fermentation, could improve health-promoting properties but also affect (potentially negatively) textural attributes of set yogurts.

## 2. Materials and Methods

### 2.1. Extraction of Crude Water Soluble Polysaccharides

Crude water soluble polysaccharides (cWSP) were extracted from hot air-dried and powdered *P. ostreatus* fruiting bodies according to the method described by Radzki et al. [38]. Briefly, the isolation procedure included the following steps: the elimination of low molecular weight compounds with 80% ethanol (at 80 °C), hot water extraction (at 120 °C), alcohol precipitation, double alcohol rinsing, and freeze-drying. Between the applied procedures, a centrifugation technique (4200× *g*) was applied to separate fluid from sediments. The extraction yield was calculated and expressed as a percentage of the mushroom dry weight.

### 2.2. Chemical Characterization of Crude Polysaccharides

Chemical characterization of the isolated cWSP involved determining the total carbohydrates, protein and phenolics. The content of carbohydrates was determined via the phenol–sulfuric acid method [39]. The sample (0.6 mL) was dissolved in water and mixed with phenol (0.6 mL, 5% *w*/*v*) and concentrated sulfuric acid (3 mL). After 40 min of incubation, the absorbance was measured at 490 nm, using a Helios Gamma spectrophotometer (Thermo, Waltham, MA, USA). Glucose was used to construct the calibration curve. The protein level was quantified with Coomassie Brilliant Blue reagent (Merck, Darmstadt, Germany) according to the Bradford method [40]. The dissolved sample (50 µL) was mixed with 2.5 mL of freshly prepared Bradford reagent and incubated for 6 min at room temperature. The absorbance was read at 595 nm (as above) and bovine serum albumin was used as standard. The total phenolics content of cWSP was determined according to Singleton and Rossi [41]. The dissolved cWSP (0.1 mL) was mixed with 0.1 mL of Folin Ciocalteu’s reagent (POCH, Gliwice, Poland) (ten times diluted), 0.1 mL of Na_2_CO_3_ (20% (*w*/*v*)) and water (0.7 mL). The mixtures were incubated for 90 min at room temperature and measured spectrophotometrically at 725 nm (as above). Different concentrations of dissolved in water gallic acid were used to construct the calibration curve. The antioxidant capacity of the cWSP solution was determined via the FRAP and ABTS method, as described in Section 2.7.

### 2.3. Bacterial Culture

Lactic acid fermentation of milk was performed with two commercially available starter cultures, Beaugel 1 (Yog1) and Beaugel 2 (Yog2), (Coquard, Villefranche Sur Saône, France) which contain *Streptococcus thermophilus* and *L. delbrueckii* ssp. *bulgaricus* strains. The cultures are dedicated to the production of yogurts and are sold as freeze-dried biomass. Each package of starter culture is dedicated to the fermentation of 5 L of milk.

### 2.4. Preparation of Reinforced Yogurts and Experiment Design

The samples (30 mL) of regenerated (13% water solution) skimmed milk powder (OSM Krasnystaw, Krasnystaw, Poland) were supplemented by the addition of freeze-dried cWSP. Five concentrations of cWSP were applied in the experiment: 0.1%, 0.2%, 0.3%, 0.4% and 0.5%, whereas samples of milk without any addition of cWSP were used as the control variant. All samples of prepared milk variants were pasteurized at 80 °C for 30 min in a water bath DEST-25 696 (Destiller, Rzeszów, Poland), cooled to the temperature of 42 °C and inoculated individually, applying one of the tested starter cultures (according to the manufacturer’s instructions). Afterwards, the samples were thoroughly mixed and transferred (30 mL) into small sterile polypropylene beakers, covered with aluminum foil and subjected to fermentation (6 h, 42 °C) using the BF 115 heating chamber (Binder, Tuttlingen, Germany). After incubation, the obtained set yogurts were stored for 24 h at 5 °C prior to physicochemical analyses.

### 2.5. Acidity and Total Soluble Content Measurements

Acidity was measured with a SevenCompact S210 pH meter (Mettler Toledo, Greifnsee, Switzerland Total soluble solids (TSS) were measured with a DR-301-95 refractometer (Krüss, Hamburg, Germany) and expressed in Brix (Bx) degrees.

### 2.6. Textural Properties and Syneresis

Textural properties were measured with a TA-XT2i texture analyzer (Stable Microsystem, Godalming, UK) equipped with a cylindrical probe (diameter, 10 mm), according to the method described by Gustaw [42]. The speed of crosshead was set at 1 mm/s during the penetration test. Three texture parameters (hardness, gumminess and cohesiveness) were determined and two of them (hardness and gumminess) were expressed in N.

Syneresis determination was based on the weight method described previously [43]. After 24 h, the gels were removed from the beakers, transferred to a metal sieve and drained. The released whey was collected and weighed. The level of syneresis was expressed as the amount of drained liquid (g) per 100 g of sample.

### 2.7. Antioxidant Capacity

Ferric-reducing antioxidant power (FRAP) assay was performed to determine the antioxidant activity of yogurts and the obtained cWSP. The methodology described by Benzie and Strain [44] was employed. The samples (0.1 mL) were mixed with 1.9 mL of freshly prepared FRAP reagent (the mixture of 300 mM acetate buffer (pH 3.6) was mixed with 10 mM 2,4,6-tripyridyl-striazine (TPTZ) solution in 40 mM HCl and 20 mM FeCl_3_∙6H_2_O (10:1:1 ratio). After incubation at 37 °C (for 15 min), the absorbance was read at 593 nm using a Helios Gamma UV-Vis spectrometer (Thermo, Waltham, MA, USA). The quantification of FRAP antioxidant capacity was performed using the calibration curve which was prepared with different concentrations of Trolox (synthetic vitamin E), and the results were expressed as Trolox µM (yogurts) or Trolox micromoles/g (cWSP).

The ability of yogurt and cWSP to neutralize free radicals was measured using the method with ABTS reagent (2,2′-azino-bis(3-ethylbenzothiazoline-6-sulfonic acid)) described by Re et al. [45]. The samples were mixed with ABTS solution which was characterized by absorbance at the level of approximately 0.7 (at a wavelength of 734 nm). The change in absorbance at 734 was measured using a Helios Gamma UV-Vis spectrometer (Thermo, Waltham, MA USA). The results were expressed as µM of Trolox (yogurts) or Trolox micromoles/g (cWSP).

### 2.8. Statistical Analysis

The yogurts, as well as all of the analysis, were performed in triplicate. The results were expressed as mean values ± standard deviation (SD). The data were assessed using unidirectional analysis of variance (ANOVA) with the level of significance set at *p* < 0.05. Tukey’s test was executed for the comparison of statistically different data. Statistical analysis was performed with the use of Statistica v. 13 software.

## 3. Results and Discussion

### 3.1. Chemical Characteristic of Water Soluble Polysaccharides

The extraction procedure which was applied in the experiment allowed us to obtain cWSP in the amount of 8%. The isolated cWSP consisted mainly of carbohydrates (60.5 ± 1.5%). Protein residue was also present and reached the level of 8.9 ± 1.7%. Some phenolics were found in small amounts and their quantity amounted to 0.8 ± 0.1%. The characteristic of cWSP was very similar to the one which was reported in previous work, where the same or very similar extraction procedure was used (carbohydrates, 58–62%; protein, 7.8–16%; phenolics, 0.77–4.5%) [38,46]. The molecular weight of cWSP was determined in previous studies with the use of size exclusion chromatography. The analysis showed that cWSP is a mixture of compounds in which the main constituents have a molecular weight of ~198 kDa, ~29 kDa and ~3 kDa [38]. The presence of protein and phenolics residue in crude polysaccharides is well known and can be explained by the fact that these compounds tend to bind to the polysaccharidic backbone either by covalent bonds or hydrophobic interactions. Proteins and phenolics which are bound to polysaccharides may play an important role in antioxidant activity [47]. Therefore, the presence of these residues is beneficial as it can improve the health-promoting potential of these macromolecules [47]. In this study, the antioxidant capacity of cWSP solution amounted to 18.4 ± 0.4 Trolox micromoles/g (FRAP) and 34.2 ± 0.8 micromoles/g (ABTS). Moreover, it was also reported that phenolics which are bound to polysaccharides are released slowly in the colon due to microbiota activity. As a result, these molecules are absorbed into bloodstream not rapidly but in a moderate way [48].

### 3.2. Physicochemical Analysis of Yogurts

**Figure 1:**
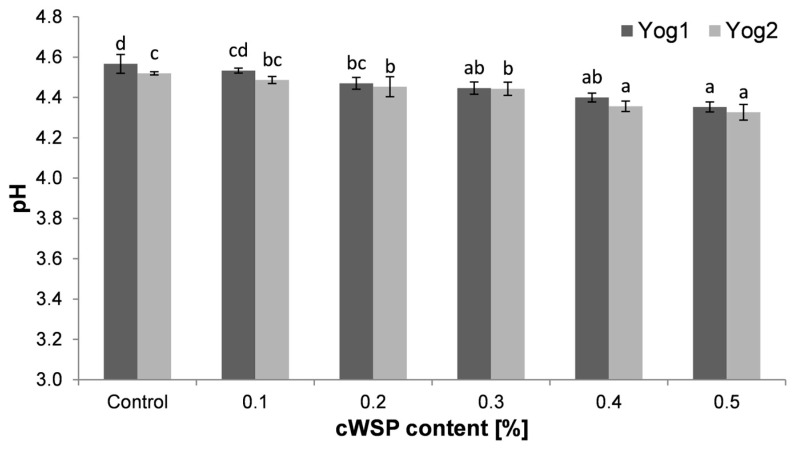
Comparison of the pH level of yogurts enriched with different concentrations of crude polysaccharides obtained from *P. ostreatus* mushroom and the control (plain yoghurt); Yog1 and Yog2 refer to different starter cultures. Bars indicate mean ± standard deviation (*n* = 3). Different letters indicate significant differences among the values according to Tukey’s test (*p* < 0.05).

The results of the pH analysis after fermentation are illustrated in Figure 1. In the case of Yog1 culture, the control sample reached pH 4.57 ± 0.05. The addition of polysaccharides caused the decrease in pH values, and the drop was proportional to the concentration of the polysaccharides. At the highest concentration (0.5%), the observed pH value amounted to 4.35 ± 0.02. These data are in accordance with previous reports, where the addition of *P. ostreatus* polysaccharidic preparations led to the increase in the total acidity of milk fermented with *A. bulgaricus* and *S. thermophilus* [49]. With regard to Yog2 culture, the pH of the control sample was slightly lower (4.52 ± 0.01) compared to Yog1. The lowest value of pH (4.33 ± 0.04) was noticed in the case of the sample with 0.5% cWSP. Similar to Yog1, a gradual drop in pH values along with the increasing concentration of cWSP was noticed. This outcome is contrary to that of Hozová et al. [35], who reported the lack of any effect of mushroom polysaccharides on pH. This discrepancy could be attributed to much lower concentrations of polysaccharides involved in the experiment.

**Figure 2:**
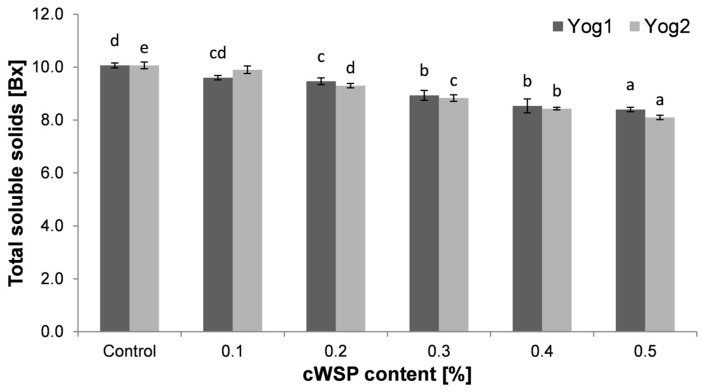
Total soluble solids (TSS) of yogurts enriched with different concentrations of crude polysaccharides obtained from *P. ostreatus* mushroom and the control (plain yoghurt); Yog1 and Yog2 refer to different starter cultures. Bars indicate mean ± standard deviation (*n* = 3). Different letters indicate significant differences among the values according to Tukey’s test (*p* < 0.05).

The data obtained from total soluble solids measurements are presented in Figure 2. The highest amount of total soluble solids was observed for the control samples (Yog1—7.97 ± 0.21% and Yog2—8.07 ± 0.12 Bx). Similar values of this parameter (7.00 ± 0.01 Bx) were observed by Bchir et al. [6], who examined yogurts made from cow’s milk. This also accords with earlier observations, which showed that the TSS of milk was at the level of 6% [50]. However, slightly higher results (9.5 ± 0.57 Bx) were obtained in another study [51]. It is apparent that along with the increase in polysaccharide quantity, the total soluble solids level decreased. Both starter cultures performed similarly in term of TSS content and there were no marked differences between them. The only exception was noticed in yogurts containing 0.5% of polysaccharides, where Yog1 showed a lower value (5.23 ± 0.12 Bx) compared to 6.1 ± 0.08 Bx of Yog2. The reason for this gradual drop in the content of TSS is not completely clear. One possible explanation is that the addition of polysaccharides stimulated the growth of bacteria, which, in turn, used sugars more efficiently [24]. Bchir et al. [6], on the other hand, noticed that the Brix values of yogurts increased with addition of sucrose-rich dehydrated pomegranate seeds.

### 3.3. Textural Properties and Syneresis

**Figure 3:**
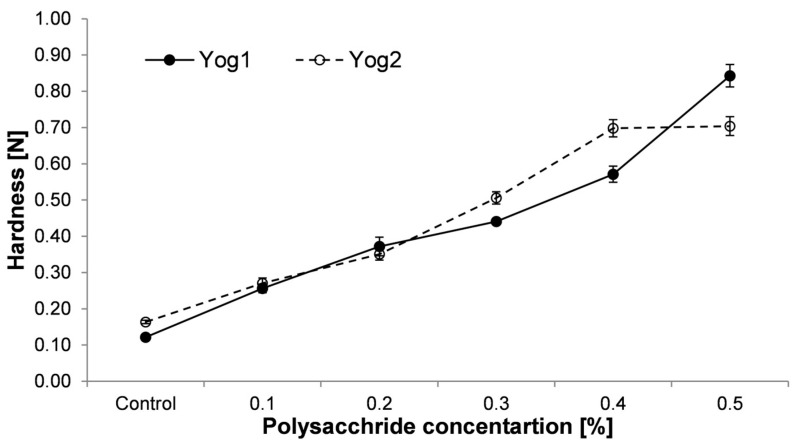
Hardness of yogurts enriched with different concentrations of crude polysaccharides obtained from *P. ostreatus* mushroom and the control (plain yoghurt); Yog1 and Yog2 refer to different starter cultures. Error bars indicates standard deviation (*n* = 3).

The value of hardness parameter represents the peak of compression force during the first compression cycle [52]. In the context of food intake, it is a force that is used by teeth (or tongue and palate in the case of semi-solids) to compress food [53]. The addition of *P. ostreatus* cWSP markedly influenced the hardness of the obtained gels, as can be seen in Figure 3. The values of this parameter raised with the increasing concentration of cWSP, regardless of the starter culture used. In the case of Yog2 culture, the plateau was reached at a concentration of 0.4%, and no further change was observed at a concentration of 0.5%. On the contrary, a rapid increase in hardness was observed in the case of yogurts fermented with Yog1 culture containing 0.5% of polysaccharides. The proportional increase in hardness to concentration of cWSP supports earlier findings by Bahrami et al. [54]. These authors reported that barley β-glucans and xanthan gum (which are both, similarly to cWSP, high molecular weight water-soluble polysaccharides) added to yogurts resulted in increased firmness compared to the control sample, which was without the addition of polysaccharides. Moreover, this study supports evidence from another observation, where crude polysaccharides from *Auricularia cornea* were applied to yogurts [31]. As was reported, the addition of polysaccharides resulted in higher firmness compared to normal yoghurt. A similar effect was observed by Kaur and Riar [55], who reported that barley β-glucans increased the firmness of yogurts. However, in contrast to the present study, Gustaw [42] noticed that the concentration of oat β-glucans, which was higher than 0.1%, resulted in a significant drop in the hardness level of yogurts. Similarly, other study reported that the hardness of exopolysaccharides (EPS)-fortified yogurts increased up to a certain concentration of EPS and then decreased [56]. Furthermore, in another study, it was observed that polysaccharides extracted from *Ganoderma lucidum* mushroom caused decreased firmness in yogurt compared to the control samples [57].

**Figure 4:**
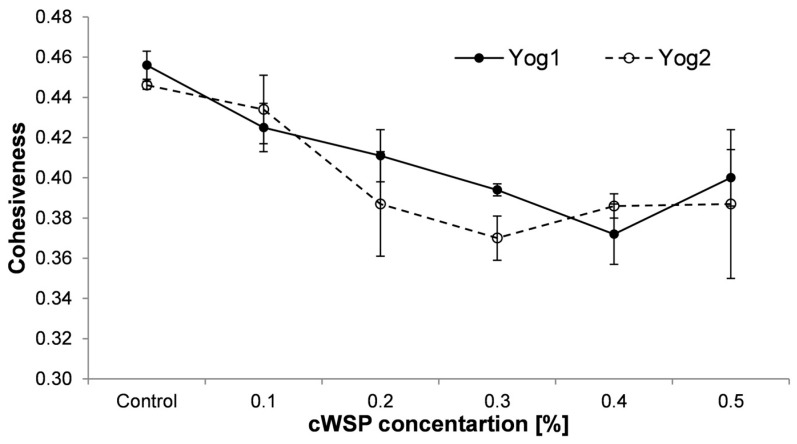
Cohesiveness of yogurts enriched with different concentrations of crude polysaccharides obtained from *P. ostreatus* mushroom and the control (plain yoghurt); Yog1 and Yog2 refer to different starter cultures. Error bars indicates standard deviation (*n* = 3).

Cohesiveness is a parameter which measures the ratio of the positive force area from the second compression to the positive force area from the first compression [52]. This parameter corresponds to force, which integrates all constituents of the product as a whole. The values of cohesiveness are presented in Figure 4. They dropped along with the concentration of cWSP, reaching minimum values at 0.4% (Yog1—0.372 ± 0.015) and 0.3% (Yog2—0.37 ± 0.011). Then, after reaching the minimum, a slight increase in this parameter was observed (0.4 ± 0.014 and 0.387 ± 0.037, respectively, for Yog1 and Yog2). A similar finding was also reported by Wang et al. [31], who noticed lower cohesiveness of yogurt which was enriched with *A. cornea* polysaccharides. Likewise, the addition of *G. lucidum* polysaccharides resulted in decreased cohesiveness [57]. The decrease in the cohesiveness parameter may be the consequence of forming gels of lower integrity and can be associated with increased syneresis [58,59].

**Figure 5:**
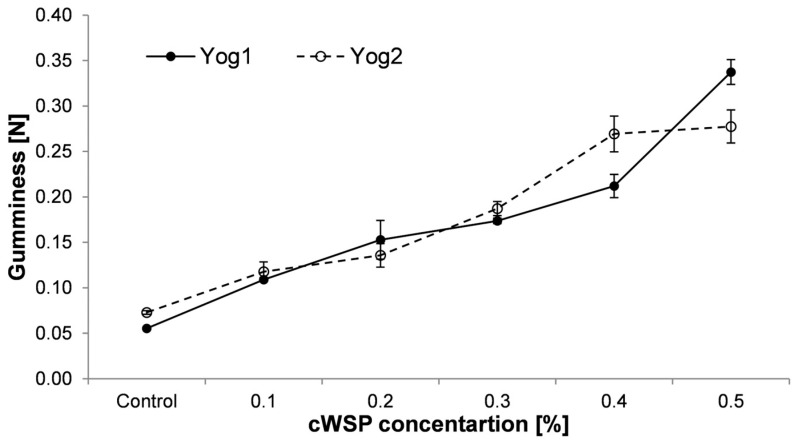
Gumminess of yogurts enriched with different concentrations of crude polysaccharides obtained from *P. ostreatus* mushroom and the control (plain yoghurt); Yog1 and Yog2 refer to different starter cultures. Error bars indicates standard deviation (*n* = 3).

With regard to gumminess, this parameter is calculated as it is defined: hardness multiplied by cohesiveness [52]. As can be seen in Figure 5, it was observed that its value increased along with polysaccharide concentration in yogurts prepared with both types of starter culture. The highest gumminess values were recorded in the samples containing 0.5% of polysaccharides (0.337 ± 0.014 and 0.277 ± 0.018 for Yog1 and Yog2, respectively). On the contrary, control samples possessed the lowest values (0.055 ± 0.001 and 0.073 ± 0.002 for Yog1 and Yog2, respectively). This outcome is contrary to that of Kaur et al. [55], who reported that the addition of barley β-glucans tends to decrease the gumminess of yogurts.

**Figure 6:**
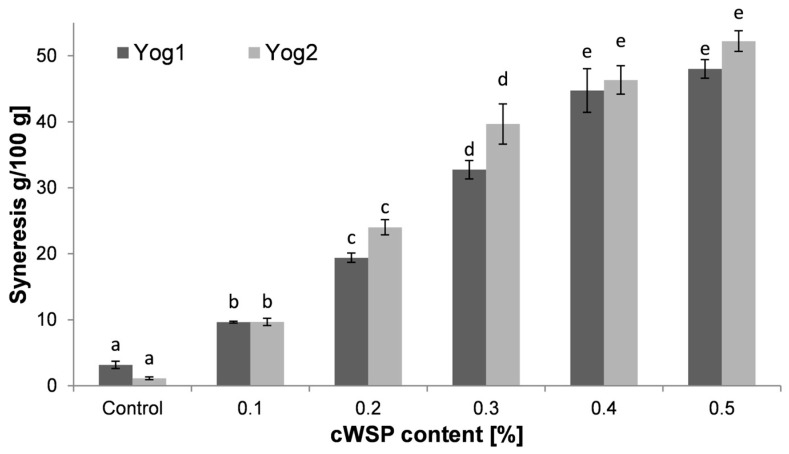
Syneresis of yogurts enriched with different concentrations of crude polysaccharides obtained from *P. ostreatus* mushroom and the control (plain yoghurt); Yog1 and Yog2 refer to different starter cultures. Bars indicate mean ± standard deviation (*n* = 3). Different letters indicate significant differences among the values according to Tukey’s test (*p* < 0.05).

Syneresis of yogurts is a process in which whey is released from the formed gel. The presence of syneresis is negatively perceived by consumers and deteriorates the acceptance of the product [60]. As can be seen from Figure 6, syneresis level was the lowest in the control samples (3.19 ± 0.55 g and 1.15 ± 0.21 g for Yog1 and Yog2, respectively). The highest values were noticed in yogurts containing 0.5% of polysaccharides (48.02 ± 1.41 g for Yog1 and 52.22 ± 1.57 g for Yog2). In general, this study showed that syneresis increase was proportional to the content of polysaccharides, regardless of the starter culture used. A similar effect was noticed by Singh et al. [61], who added the same concentrations of oat β-glucans. The present findings, however, do not support another previous study in which the addition of *P. ostreatus* and *L. edodes* polysaccharides did not worsen the consistency of yogurts (although in that work, the yogurts were evaluated only organoleptically) [35]. Moreover, Al-Sahlany et al. [10] noticed that the presence of β-glucans extracted from yeast reduced syneresis. As can be seen from Figure 3 and Figure 6, the level of syneresis was proportional to the hardness values. This is in accordance with previous results, where researchers used as an additive carrageenan and glukomannan [62] or whey preparations [63]. Singh et al. [61] concluded that adding barley β-glucans to yogurts at a level higher than 0.3% leads to excessive syneresis (higher than ~20%) and significant deterioration of product quality. This study shows that *P. ostreatus* cWSP causes even higher syneresis compared to barley β-glucans; therefore, the use of these molecules seems to be limited in the production of set-type yogurts.

### 3.4. Antioxidant Activity of Yogurts

**Figure 7:**
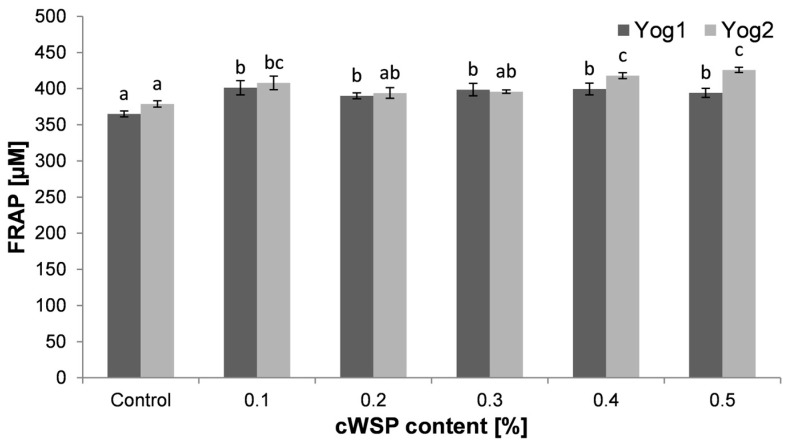
Ferric-reducing antioxidant power (FRAP) of yogurts enriched with different concentrations of crude polysaccharides obtained from *P. ostreatus* mushroom and the control (plain yoghurt); Yog1 and Yog2 refer to different starter cultures. Bars indicate mean ± standard deviation (*n* = 3). Different letters indicate significant differences among the values according to Tukey’s test (*p* < 0.05).

Ferric-reducing antioxidant power (FRAP) is an assay commonly used to measure the total antioxidant activity of compounds present in the food matrix. The method is based on the transfer of electrons from the antioxidant to ferric (Fe^3+^) ions, causing the reduction to ferrous (Fe^2+^) ions [64]. The results on antioxidant activity, measured by the FRAP method, are presented in Figure 7. The lowest values were recorded for both control samples (Yog1, 365 ± 4 µM and Yog2, 379 ± 4 µM). These results are barely distinguishable from the previous study, in which the same methodology for the determination of the antioxidant activity of plain yogurts was applied [43]. The antioxidant activity of plain yogurts results from the presence of different compounds which naturally exist in milk, i.e., peptides, proteins, amino acids or vitamins [65]. Additionally, lactic acid fermentation may improve antioxidant capacity due to the digestion of protein and releasing peptides, showing stronger antioxidant effect [66]. The samples which contained added polysaccharides displayed slightly higher antioxidant activity for both starter cultures. The highest value was observed in Yog2 containing 0.5% of polysaccharides. The observed increase in antioxidant activity could result from the presence of polysaccharides which exert antioxidant potential. Such potential of *P. ostreatus* polysaccharides was reported previously [38,67]. However, according to some studies, this potential is based on non-sugar residues like phenolics or proteins [68,69]. Fernandes and Coimbra [70] stressed that alcoholic precipitation of polysaccharides, the common step during the extraction procedure, leads to the entrapment of phenolics inside phenolic/carbohydrate complexes. Moreover, microbial fermentations can even enhance the antioxidant capacity of crude polysaccharides. According to Sinaga et al. [71], this effect can be explained by the fact that during fermentation, more phenolics are released from polysaccharides, which leads to the increase in antioxidant potential. Additionally, it was demonstrated that lactic acid bacteria *Lactobacillus plantarum* is capable of fermenting polysaccharides, which, in turn, leads to the increase in antioxidant potential of these molecules [72].

**Figure 8:**
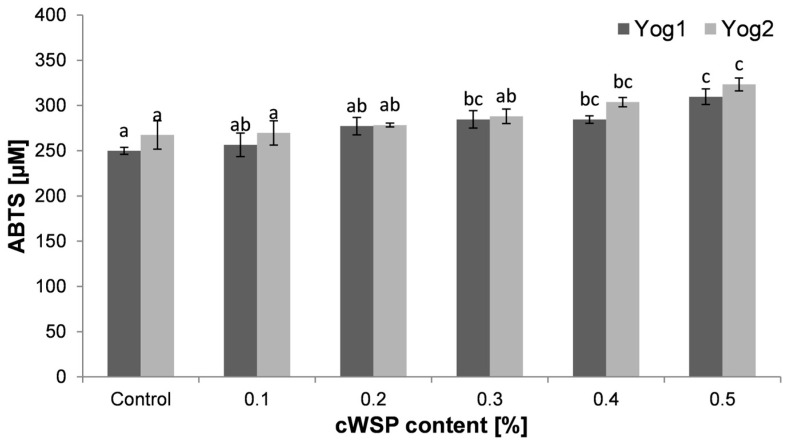
Antioxidant capacity towards ABTS radicals exerted by yogurts enriched with different concentrations of crude polysaccharides obtained from *P. ostreatus* mushroom and the control (plain yoghurt); Yog1 and Yog2 refer to different starter cultures. Bars indicate mean ± standard deviation (*n* = 3). Different letters indicate significant differences among the values according to Tukey’s test (*p* < 0.05).

Similar to the FRAP method, in the assay with ABTS, the transfer of electrons takes place during the reaction (from antioxidants to ABTS radicals). However, these two methods may produce slightly different results, and thus, usually more than one antioxidant assay is performed [73]. The ability to neutralize ABTS radicals is shown in Figure 8. As with the FRAP method, the control samples showed the lowest antioxidant potential (Yog1, 250 ± 4 μM and Yog2, 267 ± 16 μM). However, the obtained values were lower than those obtained by de Carvalho et al. [43]. Along with the increase in the concentration of cWSP in yogurts, an increase in antioxidant properties was observed. The highest levels were found at a concentration of 0.5% (310 ± 9 μM and 323 ± 7 μM, respectively, for Yog1 and Yog2). The results obtained using the ABTS method were similar to those obtained with the FRAP method, and the correlation between these two antioxidant assays can be described as strong (r = 0.7). In both methods, differences were noticed between the two starter cultures. According to Fardet and Rock [74], different bacterial strains show different proteolytic activity, which may lead to the release of a diverse quantity of antioxidant peptides.

## 4. Conclusions

The present study was designed to determine the effect of crude polysaccharides obtained from *P. ostreatus* fruiting bodies on the antioxidant and physicochemical properties of set-type yogurts. The addition of these bioactive high molecular weight molecules can improve the health-promoting properties of the enriched yogurts. Examination revealed that the fortification of yogurts can increase antioxidant potential. However, the current study is limited due to the lack of in-depth analysis of crude polysaccharides applied in the study. Detailed information on these molecules could provide additional insight into the textural properties and gel formation of yogurts. Further research could also be conducted to examine other potential bioactivities of yogurts related to the presence of mushroom-derived polysaccharides (for example: hypocholesterolemic, antitumor, immunomodulating and prebiotic). Moreover, the precise determination of compounds which are responsible for antioxidant capacity remains to be elucidated. The addition of crude polysaccharides may also potentially affect the fermentation process by stimulating the growth of lactic acid bacteria and as a result, speed up the process. However, a significant level of syneresis combined with a high hardness of gels and lowered cohesiveness was reported at the higher concentrations of crude polysaccharides (>0.2%). Therefore, this study strengthens the idea that the modification of these parameters can limit the use of such macromolecules in the production of set-type yogurts.

## Data Availability

The data presented in this study are available on request from the corresponding author.
